# 4014 Results of a Formative Evaluation of the Cardiopulmonary Vascular Biology (CPVB) Center of Biomedical Research Excellence (COBRE)

**DOI:** 10.1017/cts.2020.243

**Published:** 2020-07-29

**Authors:** Judy Kimberly, Sharon Rounds, Elizabeth O. Harrington, Susan McNamara

**Affiliations:** 1 Brown University; 2 Ocean State Research Institute

## Abstract

OBJECTIVES/GOALS: Results of a formative evaluation of the CardioPulmonary Vascular Biology (CPVB) COBRE will be presented. Of interest were the quality of the overall program, satisfaction with training, mentoring, and services offered, mechanisms for communication, and effectiveness of the collaboration between junior investigators and their mentors. METHODS/STUDY POPULATION: Integral to this evaluation was the creation of questionnaire for junior investigators to complete that addressed four domains: 1) relationship with their mentor, 2) research self-efficacy, 3) administrative and specialty cores value, and 4) satisfaction with events and operations of the COBRE. The two co-principal investigators, program manager, and evaluator developed the 34 items comprising this instrument. The questionnaire was administered online and all eight of the current junior investigators completed the questionnaire. RESULTS/ANTICIPATED RESULTS: Participants were mostly satisfied with the mentoring they were receiving and the operational services of the Administrative and Lab Cores. In terms of training preparedness, these participants felt they were not as prepared as they would like for making adequate progress as an academician and did not feel prepared for managing a lab. Interestingly, these participants felt they were most prepared to develop collaborations with scholars and professionals from other disciplines, but stated they felt they were not as prepared in their abilities to build scientific collaborations. DISCUSSION/SIGNIFICANCE OF IMPACT: Because a primary foci of COBRE grant mechanisms is the development of junior level investigators, evaluating their skills, mentoring experiences, and the usefulness of services is paramount to the sustainability and collaborative research environments of COBREs. This evaluation serves as a model for other COBREs as a tool for measuring this goal.

